# Cross aldol OPAL bioconjugation outcompetes intramolecular hemiaminal cyclisation of proline adjacent N-terminal α-oxo aldehydes at acidic pH†

**DOI:** 10.1039/d3ra08776j

**Published:** 2024-01-24

**Authors:** Afzaal Tufail, Saeed Akkad, Natasha E. Hatton, Nicholas D. J. Yates, Richard J. Spears, Tessa Keenan, Alison Parkin, Nathalie Signoret, Martin A. Fascione

**Affiliations:** a Department of Chemistry, University of York Heslington York YO10 5DD UK martin.fascione@york.ac.uk; b Hull York Medical School, University of York YO10 5DD UK nathalie.signoret@york.ac.uk

## Abstract

Novel methods to construct small molecule–protein bioconjugates are integral to the development of new biomedicines for a variety of diseases. C–C linked bioconjugates are increasingly desirable in this application due to their *in vivo* stability and can be accessed through cross aldol bioconjugation of reactive α-oxo aldehyde handles easily introduced at the N-terminus of proteins by periodate oxidation. We previously developed an organocatalyst-mediated protein aldol ligation (OPAL) for chemical modification of these reactive aldehydes, but the efficiency of this method was limited when a proline residue was directly adjacent to the N-terminus due to intramolecular hemiaminal formation. Herein we explore the competition between this cyclisation and the OPAL modification and demonstrate bioconjugation can be favoured through use of acidic pH for both oxidation and OPAL, and optimisation of reaction conditions and organocatalyst. We then showcase the utility of this acidic-OPAL in modification of the cholera toxin B-subunit (CTB), a homo-pentameric protein of biomedical promise.

## Abstract 2

Bioconjugation at N-terminal α-oxo aldehydes is challenging when proline is adjacent due to competing cyclisation. In this study we show aldol bioconjugations can outcompete this cyclisation at acidic pH in modifications of peptides and proteins.

## Graphical abstract

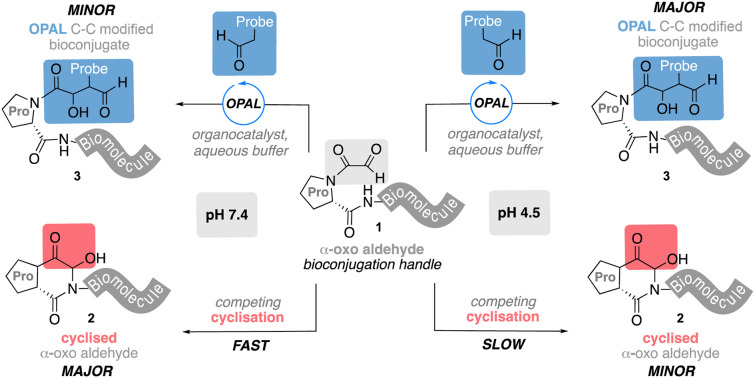

## Introduction

**Fig. 1:**
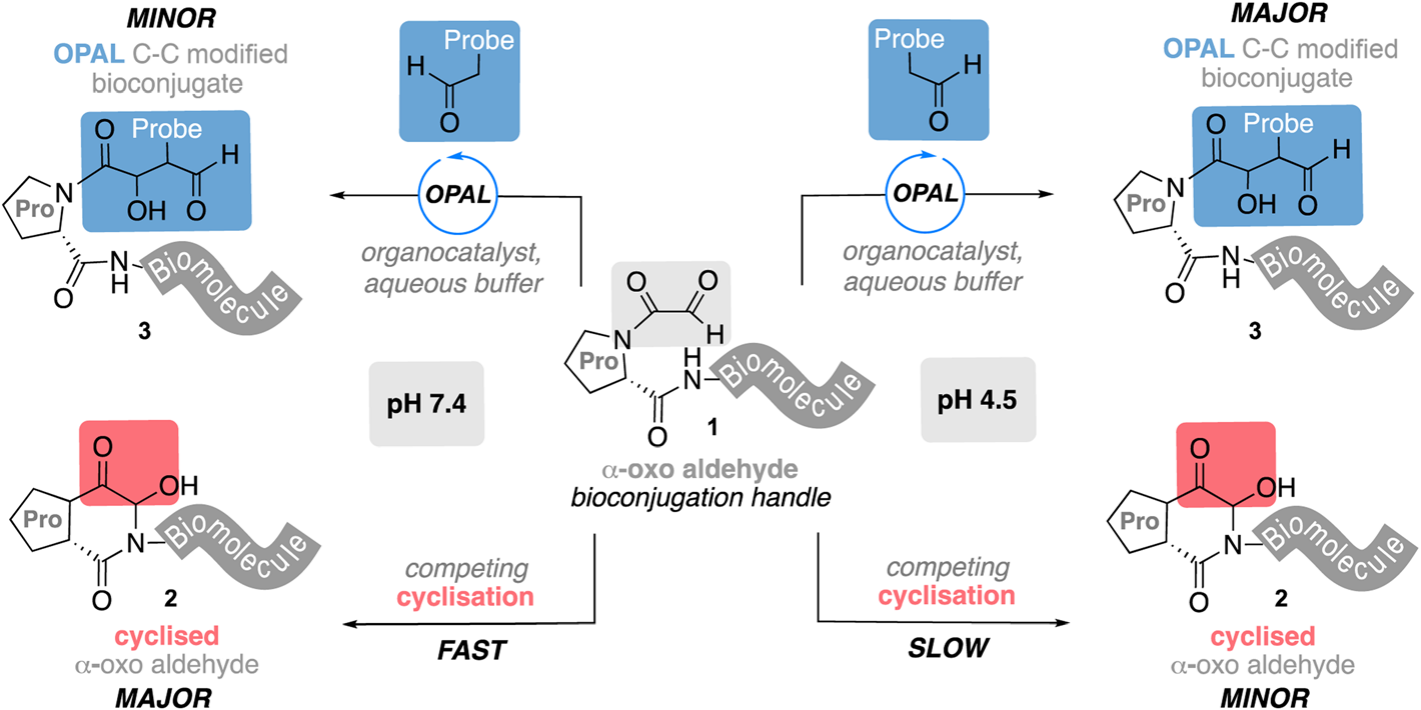
Competing OPAL aldol bioconjugation and intramolecular cyclisation of proline adjacent N-terminal α-oxo aldehydes at different pH.

Bioconjugation is a term used to collectively describe chemical strategies^1,2^ for linking small molecules to biomolecules *via* covalent bonds and has emerged as a powerful tool for augmenting their native functionality and application. Protein bioconjugates have proved particularly effective in the field of biomedicine, where conjugation of polyethyleneglycol to proteins has been shown to reduce the immunogenicity and extend the half-life of biologic drugs,^3^ and chemical ligation of small molecule warheads to antibodies has underpinned the development of cancer targeting antibody–drug conjugates (ADCs).^4,5^ Advances in this field have also aided the construction of conjugate vaccines, a safer alternative to live vaccines,^6,7^ which are composed of biomolecule antigens such as glycans often covalently linked to carrier proteins through bioconjugation chemistry.^8^ The non-toxic cholera toxin B-subunit (CTB)^9^ is one such candidate carrier protein which binds to GM1,^10,11^ a glycosphingolipid presented on a variety of human cells, and can enhance the host immune response to a vaccine antigen.^12^ CTB exists as a homo-pentameric protein, a characteristic that enables multivalent presentation of biomolecules on its surface and one which has also been exploited in the development of novel therapeutics.^13,14^ Central to this goal is the ability to site-selectively modify CTB, and many efforts to this end have focused on the use of reactive α-oxo aldehyde handles,^15^ which can be introduced at the N-terminus of proteins by mild sodium meta-periodate oxidation^13^ of an N-terminal serine, or threonine as in CTB. Although highly effective, the modification of N-terminal α-oxo aldehydes has predominantly relied on aldehyde–heteroatom coupling chemistry^15^ which affords linkages that have proved to be unstable *in vivo*, with damaging consequences.^16^ This has motivated researchers to explore new approaches for the synthesis of more stable bioconjugates, recently leading to the development of methods for the construction of stable C–C bonds^17–23^ using protein aldehydes as reaction partners.^24,25^ Our own work in this field has included the development of an organocatalyst-mediated protein aldol ligation (OPAL)^26^ that efficiently affords a C–C linkage at neutral pH through cross aldol coupling of small molecule aldehyde donors and proteins bearing α-oxo aldehyde acceptors. However, when we attempted to extend these OPAL conditions to CTB bearing an α-oxo aldehyde we were unsuccessful, even when using higher organocatalyst and aldehyde donor loading. Notably CTB has a proline residue adjacent to the installed N-terminal aldehyde handle, which in pioneering early studies by Rose and co-workers^27^ was shown to undergo intramolecular cyclisation to an unreactive hemiaminal (which has the same molecular mass as the aldehyde). We hypothesized cyclisation of this α-oxo aldehyde may compete with OPAL modification of the protein at neutral pH, accounting for the challenges encountered during bioconjugation. Herein we use a model peptide system to explore this hypothesis and demonstrate that when a proline is adjacent to an N-terminal α-oxo aldehyde handle 1 the fast, unproductive intramolecular cyclisation does indeed outcompete cross aldol OPAL bioconjugation at neutral pH 7.4 (Fig. 1). However, formation of the unreactive hemiaminal 2 is slower at acidic pH 4.5 and productive C–C bioconjugation can be accelerated at this pH to drive product 3 formation through judicious choice of OPAL organocatalyst, conditions which we subsequently showcase in the aldol modification of CTB.

## Results and discussion

**Fig. 2:**
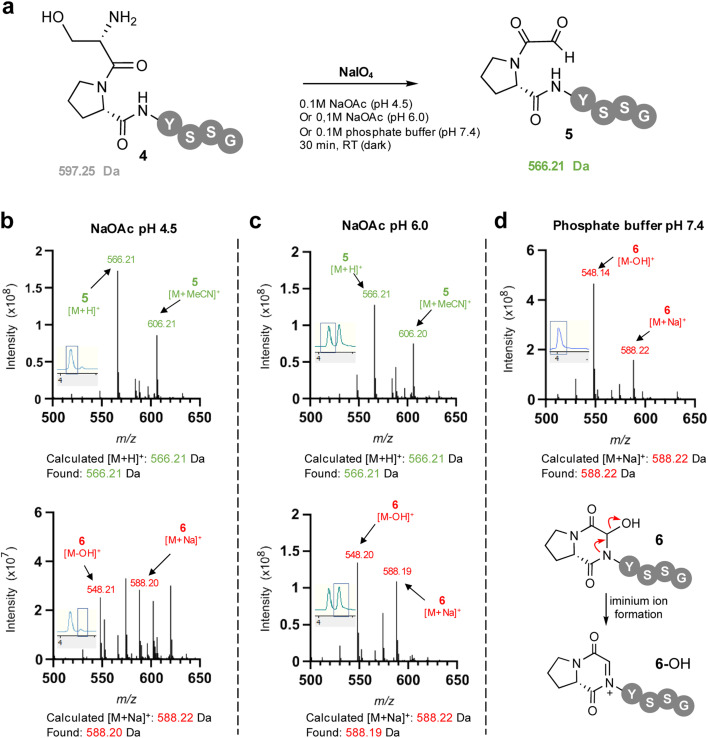
(a) Sodium meta-periodate oxidation of the N-terminal serine in SPYSSG 4 to afford the desired α-oxo PYSSG 5 at different pH. (b) Positive ion mass spectrum of selected peaks in RP-LCMS 210–400 nm UV chromatogram (chromatogram trace inset) following oxidation of 4 in pH 4.5 NaOAc buffer for 30 min. (c) Positive ion mass spectrum of selected peaks in RP-LCMS 210–400 nm UV chromatogram (chromatogram trace inset) following oxidation of 4 in pH 6.0 NaOAc buffer for 30 min. (d) Positive ion mass spectrum of selected peaks in RP-LCMS 210–400 nm UV chromatogram (chromatogram trace inset) following oxidation of 4 in pH 7.4 phosphate buffer for 30 min, indicating formation of only hemiaminal 6 which is detected in the mass spectrum as characteristic charged iminium ion 6-OH.

To facilitate study of the intramolecular hemiaminal formation we used solid phase peptide synthesis (Fig. S1†) to assemble a test-bed hexapeptide SPYSSG 4, containing a proline adjacent to the N-terminal serine to be oxidised to an α-oxo aldehyde. Cognisant that the cyclisation may occur rapidly once the α-oxo aldehyde was formed we initially focused on identifying the optimum pH for the periodate oxidation of the peptide N-terminus (Fig. 2a), screening pH 4.5 and pH 6 in acetate buffer, and pH 7.4 in phosphate buffer. Following oxidation for 30 min at pH 4.5 and rapid purification on a reverse phase (RP) solid phase extraction cartridge using acetonitrile, positive-ion RP-LCMS analysis (Fig. 2b) revealed formation of two products with an UV absorbance. The first to elute was the major and desired product α-oxo-PYSSG 5 with a molecular mass of 565 Da and characteristic [M + H]^+^ and [M + MeCN]^+^ adducts in the mass spectrum of this UV chromatogram peak (Fig. 2b inset). A minor product eluted after 5 and was identified as the hemiaminal product 6 of intramolecular cyclisation, which has the same molecular mass as α-oxo aldehyde 5, but is detected in the mass spectrum as a characteristic positively charged iminium ion 6-OH which has a molecular mass of 548 Da, and is accompanied by a [M + Na]^+^ adduct of the hemiaminal 6. This indicated periodate oxidation at pH 4.5 predominantly yielded the α-oxo aldehyde product, however equivalent oxidation at pH 6 (Fig. 2c) instead afforded approximately equal conversion to α-oxo aldehyde 5 and hemiaminal 6, demonstrating intramolecular cyclisation is favoured at pH 6 over pH 4.5. Further evidence that the cyclisation is favoured at more neutral pH was provided by equivalent oxidation at pH 7.4 (Fig. 2d), which afforded only the cyclic hemiaminal product 6, with no evidence of any remaining α-oxo aldehyde 5. These results therefore suggested that in order to maximise conversion in any subsequent OPAL bioconjugations pH 7.4 should be avoided, as the intermolecular bioconjugation would be in competition with the intramolecular cyclisation which occurs rapidly.

**Fig. 3:**
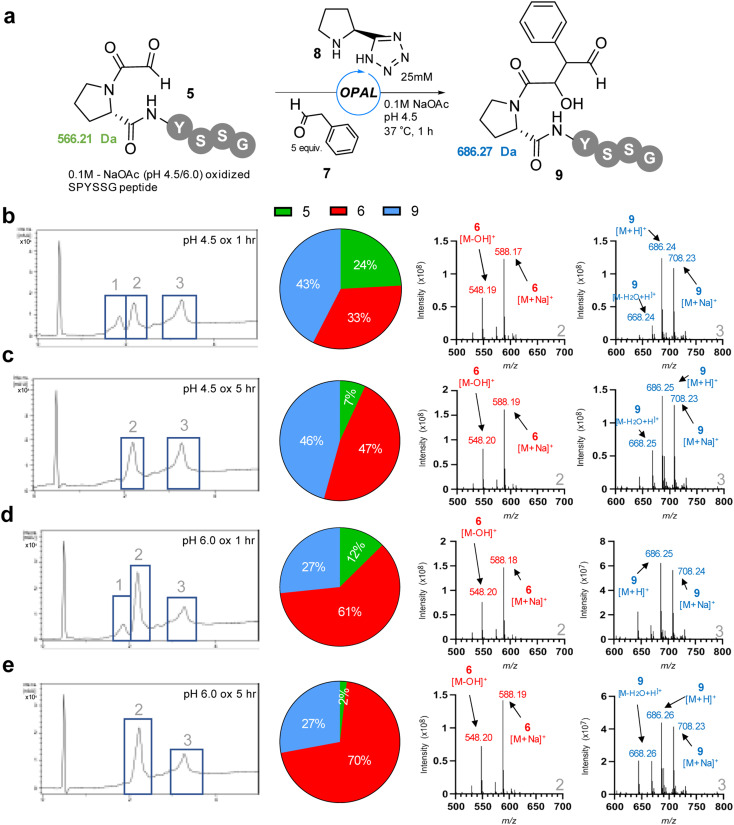
(a) pH 4.5 or pH 6.0 oxidised α-oxo-PYSSG 5 subjected to OPAL bioconjugation at pH 4.5 using 5 equiv. phenylacetaldehyde 7 and organocatalyst 8 to afford desired OPAL product 9. (b) LCMS analysis of 1 h OPAL reaction using pH 4.5 oxidised α-oxo-PYSSG 5 as a starting material. (c) LCMS analysis of 5 h OPAL reaction using pH 4.5 oxidised α-oxo-PYSSG 5 as a starting material. (d) LCMS analysis of 1 h OPAL reaction using pH 6.0 oxidised α-oxo-PYSSG 5 as a starting material. (e) LCMS analysis of 5 h OPAL reaction using pH 6.0 oxidised α-oxo-PYSSG 5 as a starting material (Left) RP-LCMS UV chromatogram. (Centre) Pie chart depicting relative conversions to hemiaminal 6 (red), OPAL product 9 (blue), or remaining starting material 5 (green). (Right) Positive ion mass spectrum of chromatogram peaks 2 (hemiaminal 6) and 3 (OPAL product 9).

We thus opted to screen new pH 4.5 conditions for the OPAL bioconjugation with 5 equivalents of aldol donor phenylacetaldehyde 7 using our previously established^26^ pH 7.4 optimal organocatalyst proline tetrazole 8 (Fig. 3), with the aim of outcompeting cyclisation at lower pH, on α-oxo-PYSSG 5. Initially using pH 4.5 oxidised 5 as the aldehyde acceptor in the OPAL reaction we observed three peaks in the RP-LCMS UV chromatogram trace after 1 h, with peak 1 identified as the remains of the α-oxo aldehyde starting material 5 in 24% relative conversion, peak 2 the undesired hemiaminal 6 in 33% conversion, and peak 3 the desired OPAL product 9 in 43% conversion (Fig. 3b). If the cyclisation was reversible at acidic pH we hypothesized we would observe increased conversion from cyclic 6 to OPAL product 9 as the reaction time was extended. However, after 5 h (Fig. 3c) although the majority of α-oxo aldehyde 5 was consumed we observed a greater relative increase in conversion to the hemiaminal 6 (47%) rather than the desired OPAL product 9. These results demonstrated cyclisation does still occur at pH 4.5 when prolonged reaction times are used. We then screened pH 6.0 oxidised 5 as the starting material in OPAL reaction at pH 4.5 to determine whether the pH at which the aldehyde was formed would have any effect on the subsequent reactivity, and unsurprisingly observed a similar trend. In the 1 h reaction (Fig. 3d) we observed an increase relative conversion of 61% to undesired hemiaminal 6, which would be expected as our oxidation results indicated an appreciable amount of α-oxo peptide had already cyclised during oxidation at pH 6.0. And once again, when the reaction was extended to 5 h (Fig. 3e), although much of the α-oxo aldehyde 5 was consumed, only a relative increased conversion to the undesired hemiaminal 6 was observed. Taken together these experiments infer that at acidic pH N-terminal cyclisation is not reversible at any appreciable rate. This observation was reinforced by performing OPAL bioconjugation at pH 4.5 using the pure cyclic hemiaminal 6 as the starting material and detecting no OPAL product formation (Fig. S2†). We therefore concluded acidic conditions were optimal for OPAL bioconjugation for these proline adjacent α-oxo aldehydes as competing cyclisation was slower in our experiments at neutral pH.

**Fig. 4:**
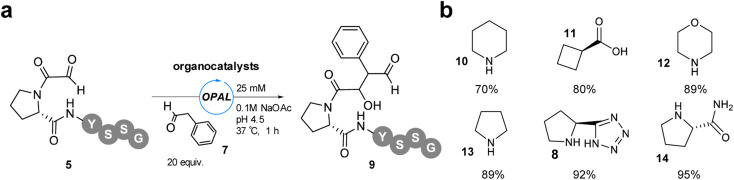
(a) pH 4.5 OPAL bioconjugation of α-oxo-PYSSG 5 using 20 equiv. phenylacetaldehyde 7 and 25 mM organocatalyst (8, 10–14). (b) Structures of organocatalysts 8, 10–14 and % conversion to product 9 when used in pH 4.5 OPAL bioconjugation.

As only a maximum 46% conversion to OPAL bioconjugation product was observed in these initial studies at pH 4.5 and extending the reaction time did not lead to increase product formation, we elected to increase the amount of aldol donor 7 used to 20 equivalents in order to outcompete the intramolecular cyclisation. With α-oxo-PYSSG 5 as the acceptor we also screened a range of potential OPAL organocatalysts to determine the most effective catalyst at acidic pH (Fig. 4). A panel of six organocatalysts 8 and 10-14 were therefore deployed at pH 4.5 for 1 h, with piperazine 10 affording the lowest conversion and (*S*)-prolinamide 14 the highest 95% conversion, marginally superior to proline tetrazole 8 which was previously identified as the optimal OPAL organocatalyst at pH 7.4.

**Fig. 5:**
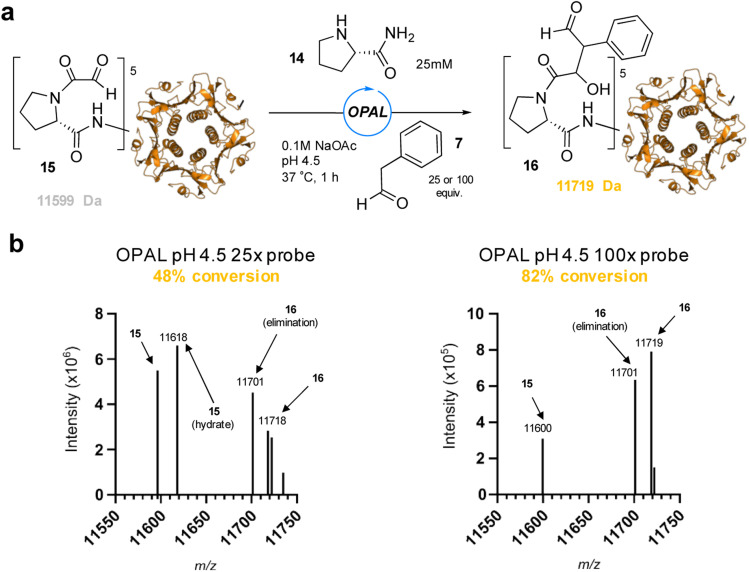
(a) pH 4.5 OPAL bioconjugation of pentameric α-oxo CTB 15 using prolinamide organocatalyst 14 and either 25 or 100 equiv. of phenylacetaldehyde 7 to afford OPAL CTB product 16. (b) Deconvoluted protein LCMS of reaction mixture following OPAL bioconjugation with either 25 equiv. of 7 (left), or 100 equiv. of 7 (right).

**Fig. 6:**
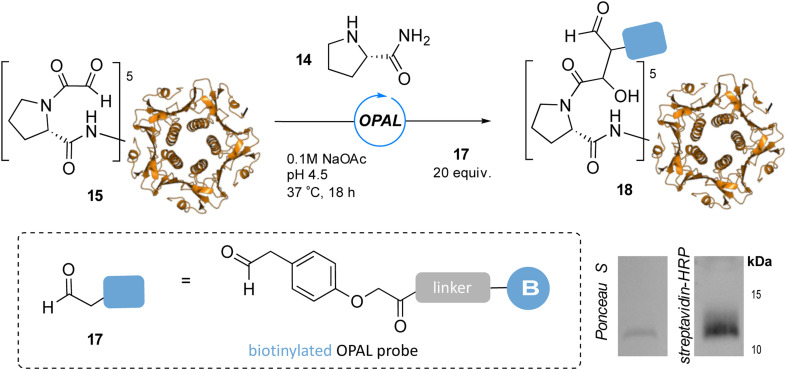
pH 4.5 OPAL bioconjugation of pentameric α-oxo CTB 15 using prolinamide organocatalyst 14 and 20 equiv. of biotin OPAL probe 17 to afford biotinylated CTB product 18, analysed following 18 h bioconjugation by Ponceau S staining and streptavidin-HRP western blot (bottom right), CTB monomer at ∼12 kDa.

To showcase the utility of these newly optimized acidic-OPAL bioconjugation conditions we returned to the challenging protein modification of pentameric CTB bearing an N-terminal α-oxo aldehyde 15 (Fig. 5). Acidic-OPAL bioconjugation was performed on 15 in the presence of organocatalyst 14 at pH 4.5 using either 25 or 100 equivalents of phenylacetaldehyde 7 donor for 1 h. Pleasingly 48% conversion to CTB OPAL product 16 using 25 equivalents of 7 was determined through protein MS characterisation of the CTB monomer, and an increased 82% conversion when using 100 equivalents of aldol donor 7. Following on from the use of this simple aldol donor we then synthesised a more complex aryl acetaldehyde containing probe 17 for biotin OPAL labelling of the CTB N-terminus (Fig. 6). Acidic-OPAL bioconjugation was again performed on α-oxo-CTB 15 using organocatalyst 14 and left for 18 h to maximise conversion to OPAL product 18 using 20 equivalents of biotin probe 17. Purification of the protein from excess complex probe proved challenging precluding protein MS characterization, but western blot analysis of the CTB monomer using streptavidin-HRP visualization confirmed successful biotinylation had taken place (Fig. 6).

## Conclusions

This work therefore demonstrates that the acidic-OPAL is applicable to the modification of complex biomolecules and may have broad use in the chemical functionalisation or immobilisation of proteins containing N-terminal adjacent prolines, or proteins that are most stable at acidic pH. Despite the substantial improvement achieved by performing OPAL chemistry at acidic pH rather than neutral pH with proline adjacent α-oxo aldehydes, cross aldol bioconjugations under these conditions still require further optimisation to maximise conversion. In future studies this could be achieved through exploration of alternative aryl acetaldehyde probe scaffolds^19^ and extensive screening of a broader panel of organocatalysts to identify those even more effective in aldol reactions in aqueous acidic conditions.^28^

## Author contributions

AT, SA, and RJS performed peptide and protein bioconjugations. RJS and TK purified proteins and NEH and NDJY performed chemical synthesis. AP, NS and MAF supervised the project. AF, NS and MAF wrote the manuscript and designed the study. All authors analysed the data and commented on the manuscript.

## Conflicts of interest

The authors declare no competing financial interest.

## Supplementary Material

RA-014-D3RA08776J-s001
