# Supplementary material for: Cross aldol OPAL bioconjugation outcompetes intramolecular hemiaminal cyclisation of proline adjacent N-terminal α-oxo aldehydes at acidic pH
Source: RSC Adv. 2024 Jan 24;14(6):3723–9. doi: 10.1039/d3ra08776j (PMC10806391; doi:10.1039/d3ra08776j)
Supplement: RA-014-D3RA08776J-s001 [file RA-014-D3RA08776J-s001.pdf]

Electronic Supplementary Material (ESI) for RSC Advances.
This journal is © The Royal Society of Chemistry 2024

Supplementary Information

Cross aldol OPAL bioconjugation outcompetes intramolecular hemiaminal cyclisation of proline adjacent N-terminal α-oxo aldehydes at acidic pH

Afzaal Tufail[1,2], Saeed Akkad[1], Natasha E. Hatton[1], Nicholas D. J. Yates[1], Richard J. Spears[1], Tessa Keenan,[1] Alison Parkin,[1] Nathalie Signoret[2]* and Martin A. Fascione[1]*

[1]Department of Chemistry, University of York, Heslington, York, YO10 5DD, UK. [2]Hull York Medical School, University of York, YO10 5DD

Supplementary Figures referenced in main text

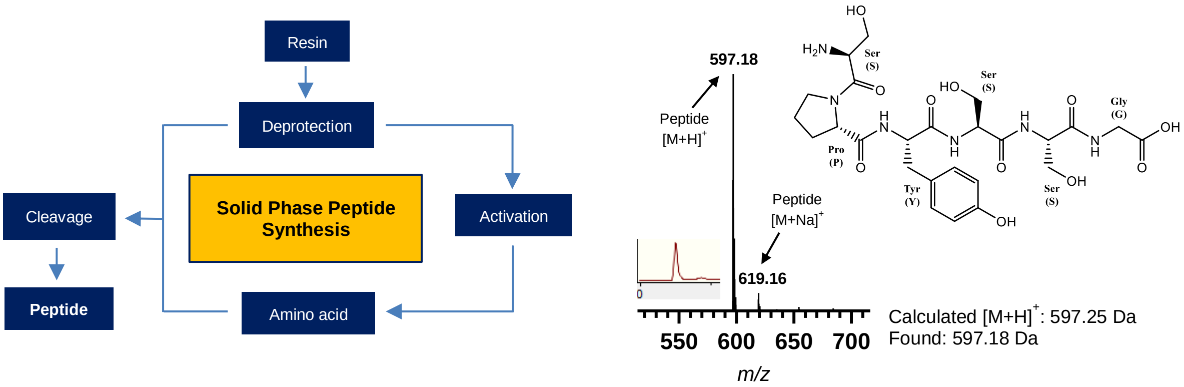

Figure S1. Synthesis of heaxapetide (SPYSSG) using solid phase peptide synthesis

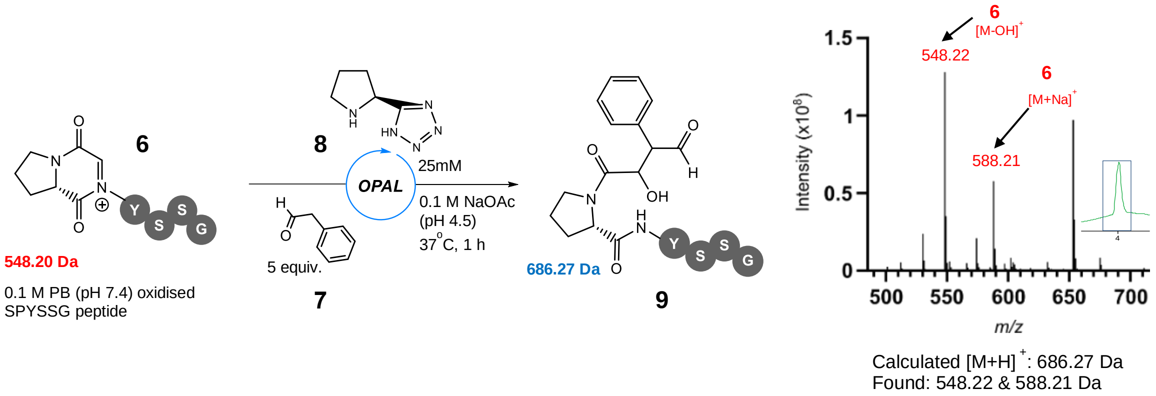

Figure S2. Acidic OPAL bioconjugation (pH 4.5) on cyclic hemiaminal (PB oxidised SPYSSG), demonstrating no OPAL product **9** formation under acidic-OPAL conditions.

Methodology

LC-MS method

High Performance Liquid Chromatography-Electrospray Ionization Mass Spectrometry (LC-MS) of peptides and OPAL products was performed using a Dionex UltiMate® 3000 Ci Rapid Separation LC system equipped with an UltiMate® 3000 photodiode array detector probing at 250–400 nm, coupled to a HCT ultra ETD II (Bruker Daltonics) ion trap spectrometer, using Chromeleon® 6.80 SR12 software (ThermoScientific), esquireControl version 6.2, Build 62.24 software (Bruker Daltonics), and Bruker compass HyStar 3.2-SR2, HyStar version 3.2, Build 44 software (Bruker Daltonics) at the Centre of Excellence in Mass Spectrometry, York. All mass spectrometry was conducted in positive ion mode. Peptide samples were eluted off an LC-18 SPE cartridge in 30% MeCN + 0.1% formic acid prior to LC-MS analysis and were chromatographically analysed using an Accucore C18, 2.6 μ (50 mm × 2.1 mm, Thermoscientific). Sample peptides were in water with 0.1% (v/v) formic acid (solvent A) and acetonitrile with 0.1% (v/v) formic acid (solvent B) mobile phase at a flow rate of 0.3 mL min−1 at RT. A multistep gradient of 13.0 min was programmed as follows: 5% B for 0 min, a linear gradient to 10% B over 1 min, a linear gradient to 30% B over 1 min, a linear gradient to 50% B over 2 min, a linear gradient to 90% over 1 min, 90% B for 5 min, linear gradient to 5% B over 1 min, 5% B for 2 min. Note that the multistep gradient finishes in 5% B in order to re-equilibrate the column. Protein samples were desalted using a PD minitrap G25 column and were analysed in 1 : 1 water : acetonitrile + 1% (v/v) formic acid. Protein samples were analysed without the use of a column at a flow rate of 0.25 mL min−1 at RT.

Synthesis of SPYSSG

SPYSSG peptide was synthesised using Fmoc solid-phase peptide synthesis (SPPS). H-Gly-2-ClTrt resin (300 mg, 0.79 mmol/g, 0.237 mmol, 200-400 mesh, Novabiochem) was weighed into an SPPS cartridge fitted with a PTFE stopcock, swollen in DMF for 30 minutes and then filtered. Each amino acid coupling for the generation of SPYSSG used the following method: DIPEA (11 eq) was added to a solution of Fmoc-protected amino acid (5 eq) and HCTU (5 eq) dissolved in the minimum volume of DMF, which was added immediately to the resin. The reaction mixture was gently agitated by rotation for 1 h and the resin was filtered off and washed with DMF (3 x 2 minutes with rotation). A solution of 20 % piperidine in DMF was added to the resin and the mixture gently agitated (5 x 2 minutes with rotation). The resin (now bearing deprotected amine functionality) was then washed using DMF (5 x 2 minutes with rotation) prior to coupling on the next amino acid. The order of the amino acids used to prepare the peptide were as follows: Fmoc-*O*-tert-butyl-Serine, Fmoc-*O*-tert-butyl-Serine, Fmoc-*O*-tert-butyl-Tyrosine, Fmoc-Proline and Fmoc-O-tert-butyl-Serine. Coupling of the proline residue required an additional coupling step. After the addition of the Fmoc-Proline, the resin was filtered and washed with DMF (3 x 5 minutes with rotation). Without Fmoc cleavage, another solution of activated Fmoc-Proline was added (1 h rotation), washed with DMF (3 x 5 minutes with rotation) and 20% piperidine in DMF (5 x 2 minutes with rotation). After coupling on the final serine residue and washing with DMF (5 x 2 minutes with rotation), the resin was washed with DCM (3 x 2 minutes with rotation) and MeOH (3 x 2 minutes with rotation). The resin was dried on a vacuum manifold and further dried on a high vacuum line overnight. 5 mL of cleavage cocktail solution (95:2.5:2.5 TFA:H_2O:triisopropylsilane) was then added to the resin and the mixture was agitated by rotation for 1 h. The reaction mixture was drained into cold Et_2O and centrifuged at 4000 x g at 4 °C until pelleted (ca 5 minutes). The supernatant was then carefully decanted and the pellet was subsequently resuspended in cold Et_2O and the centrifugation and supernatant decantation process repeated three times. The precipitated peptide pellet was then dissolved in 10% AcOH and lyophilised to obtain SPYSSG **4** as a fluffy solid (69 mg, 49%) for use without further purification, see Fig. S1 for LC-MS analysis.

SPYSSG peptide N-terminal serine oxidation

Oxidation of SPYSSG **4** to glyoxyl-PYSSG **5** was carried out by dissolving a desired amount of peptide in 1 mL of 0.1 M phosphate buffer (PB) pH 7.4 or 0.1 M NaOAc pH 4.5 or 0.1 M NaOAc pH 6.0 followed by the addition of 10 equiv. $NaIO_4$. The solution was vortexed, then allowed to sit at room temperature in the dark for 30 minutes. The reaction was loaded onto a solid phase extraction cartridge (Supelco Supelclean LC-18 SPE, 6 mL reservoir) equilibrated with water/acetonitrile. After initial washing with water, the product was eluted in 30% acetonitrile. Fractions containing pure, oxidised peptide (assessed by LC-MS) were pooled and lyophilised to give glyoxyl-PYSSG **5** which was stored at -20 °C.

Organocatalyst Protein Aldol Ligation (OPAL) reaction with SPYSSG 6

A 100 µL aliquot of a 5 mM glyoxyl-PYSSG **5** stock in 0.1 M NaOAc, or 0.1 M PB was charged with 250 uL 0.2 M NaOAc pH 4.5 and 22.5 µL DMSO. The reaction was then charged with 12.5 µL of (*S*)-5-(Pyrrolidin-2-yl)-1H-tetrazole organocatalyst from a 1 M stock solution and 2.5 µL of phenylacetaldehyde donor (1 M stock in DMSO). Reaction volume was completed to 500 µL with LC-MS water and the solution was vortexed and allowed to sit at 37 °C for indicated time with agitation (350 rpm). Conversion to the aldol products were confirmed by LC-MS.

Organocatalyst Protein Aldol Ligation (OPAL) organocatalyst screening

A 100 µL aliquot of a 5 mM glyoxyl-PYSSG stock in 0.1 M NaOAc, was charged with 250 uL 0.2 M NaOAc pH 4.5 and 15 µL DMSO. The reaction was then charged with 50 µL of organocatalysts from a 1 M stock solution and 10 µL of phenylacetaldehyde donor (1 M stock in DMSO). The pH of the different organocatalysts were adjusted to pH 4.5. Reaction volume was completed to 500 µL with LC-MS water and the solution was vortexed and allowed to sit at 37 °C for 1 h with agitation (350 rpm). Conversion to the aldol products and efficiency of organocatalysts were assessed by LC-MS.

Cholera Toxin (CTB) preparation

The protocol for obtaining this protein was adapted from a published protocol.[1] A stab from a stock of *Escherichia coli* BL21 DE23 containing a pSAB2.2 plasmid (kindly provided by Prof W. Bruce Turnbull) encoding CTB was used to inoculate LB growth media (5 mL) containing ampicillin (100 µg mL-1) and this starter culture was incubated at 37 °C for 20 h. Starter culture (1 mL) was added to LB growth media (4 x 1 L) which was then incubated at 37 °C and monitored until the OD600 reached ca. 0.5 before IPTG (1 M final concentration) was added to each flask in induce protein over-expression. The incubation was continued for 24 h at 30 °C before isolating the cells by centrifugation at 10000 x g for 10 mins. The bacterial cell pellet was discarded and the supernatant was retained. Solid ammonium sulfate was added to the supernatant (final concentration 60% w/v) and stirred for 1 h. The solution was then centrifuged at 17000 x g for 25 mins and the supernatant discarded. The protein pellet was resuspended in 0.1 M phosphate buffer and centrifuged at 15000 x g for 8 mins to remove any insoluble material. The supernatant was filtered through a 0.45 µm filter (Sartorius Minisart) and loaded onto 2 x 5 ml HisTrap FF Ni columns. Protein purification was then performed by first washing the Ni column with 0.1 M phosphate buffer containing 20 mM imidazole (pH 7, dubbed Buffer A) and then eluting bound protein with 0.1 M phosphate buffer containing 500 mM imidazole (pH 7, dubbed Buffer B) over a gradient of 0-100% Buffer B over 30 minutes using an AKTA Start (GE Healthcare Life Technologies). Protein presence in collected fractions was monitored by UV spectroscopy at 280 nm. Fractions thought to contain the desired protein were confirmed by SDS-PAGE analysis.

Cholera Toxin (CTB) oxidation

A 100 µL aliquot of an 453 µM CTB stock (in 0.1 M PB, 0.1 M NaCl, pH 7.0 was charged with 7 µL of a 66 mM L-methionine stock solution in 0.1 M PB, 0.1 M NaCl, pH 7.0, and 7 µL of a 33 mM $NaIO_4$ stock solution in 0.1 M PB, 0.1 M NaCl, pH 7.0. The solution was mixed by gentle pipetting and allowed to sit on ice in the dark for 4 minutes. The reaction was immediately purified using a PD SpinTrap G-25 desalting column eluting into 0.1 M NaOAc pH 4.5. Quantitative oxidation to glyoxyl-CTB was confirmed by LC-MS analysis.

Cholera Toxin (CTB) acidic-OPAL labelling with phenylacetaldehyde (PAA) 7

A 200 µL aliquot of a 105 µM glyoxyl-CTB stock in 0.1 M NaOAc, pH 4.5, was charged with 80 µL of 0.5 M NaOAc pH 4.5 and 18.95 µL DMSO (5% overall in reaction). The reaction was then charged with 10 µL of *S*-prolinamide organocatalyst from a stock solution (1 M in 0.1 M NaOAc pH 4.5) and 25x or 100x of phenylacetaldehyde donor **7** (1 M stock in DMSO). Reaction volume was completed to 400 µL with LC-MS water. The solution was vortexed and allowed to sit at 37 °C overnight (18 h) with agitation (350 rpm). Conversion to the anticipated aldol product were confirmed by LC-MS.

Cholera Toxin Cholera (CTB) acidic-OPAL labelling biotin probe 17[2]

A 6.56 µL aliquot of a 571 µM glyoxyl-CTB stock in 0.1 M NaOAc, pH 4.5, was charged with 10 µL of 0.5 M NaOAc pH 4.5 and 1.25 µL DMSO. The reaction was then charged with 1.25 µL of *S*-prolinamide organocatalyst from a stock solution (1 M in 0.1 M NaOAc pH 4.5) and with 20 equivalents of biotin probe **17**[2] (4 mM stock in 0.1 M NaOAc pH 4.5). The volume was completed to 50 µL with water, vortexed and allowed to sit at 37 °C overnight (18 h) with agitation (350 rpm). Conversion to the anticipated aldol product was confirmed by SDS-PAGE analysis. Reaction was desalted into 20 mM sodium phosphate buffer, 150 mM NaCl (pH 7.4). Samples were combined with 5x reducing SDS sample buffer boiled at 95 °C (10 minutes) and resolved on a 15% SDS-PAGE gel and transferred onto nitrocellulose membrane using transblot transfer system (Biorad). The membrane was blocked in TBS-Tween (0.1%) containing 5% milk, before labelling with streptavidin-HRP (Sigma)1/5000 in TBS-T milk and detecting the HRP signal on iBright CL1500 software version 1.8.0.

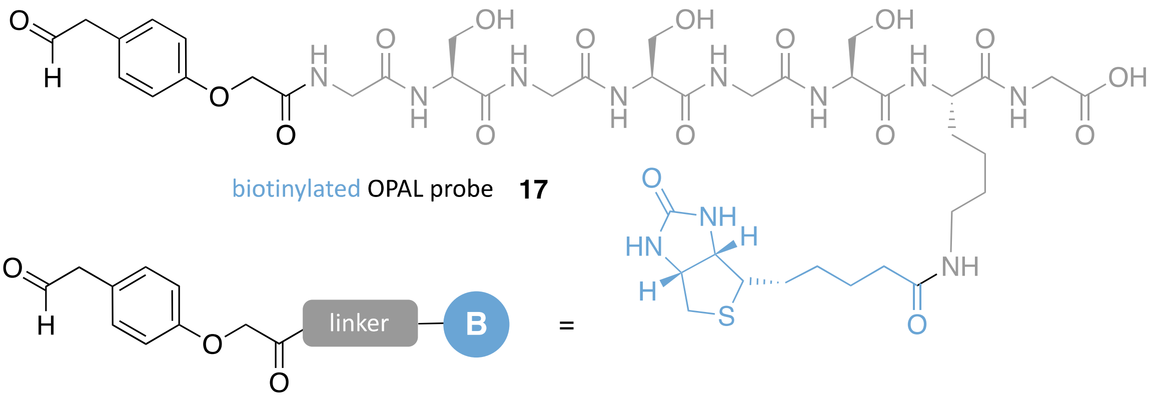

Figure S3. Structure of biotinylated OPAL probe **17**, synthesis previously reported.[2]

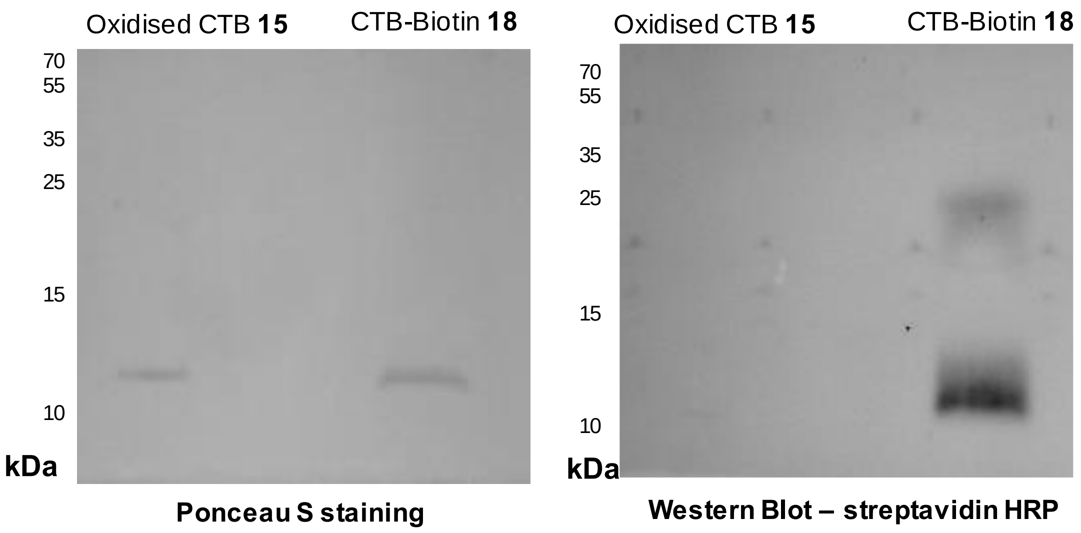

Figure S4. Ponceau S staining and streptavidin-HRP Western Blot visualisation of oxidized CTB **15** and CTB-biotin **18**, following acidic-OPAL modification using 20 equivalents of biotin probe **17.** CTB monomer at ~12 kDa, putative CTB oligomers at ~25 kDa.

LC-MS chromatograms and spectra

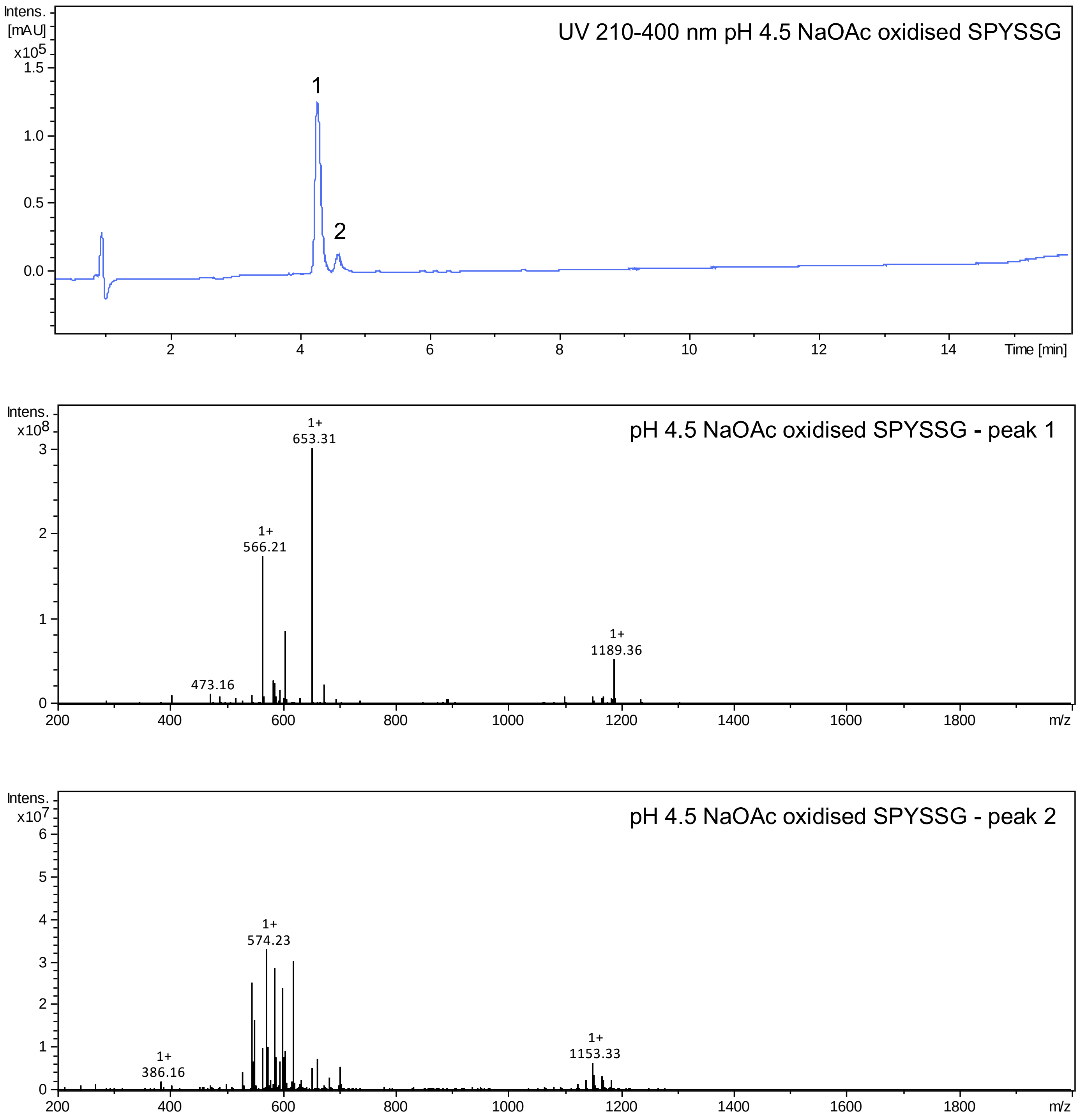

Figure S5. Full LCMS analysis of pH 4.5 NaOAc oxidised SPYSSG

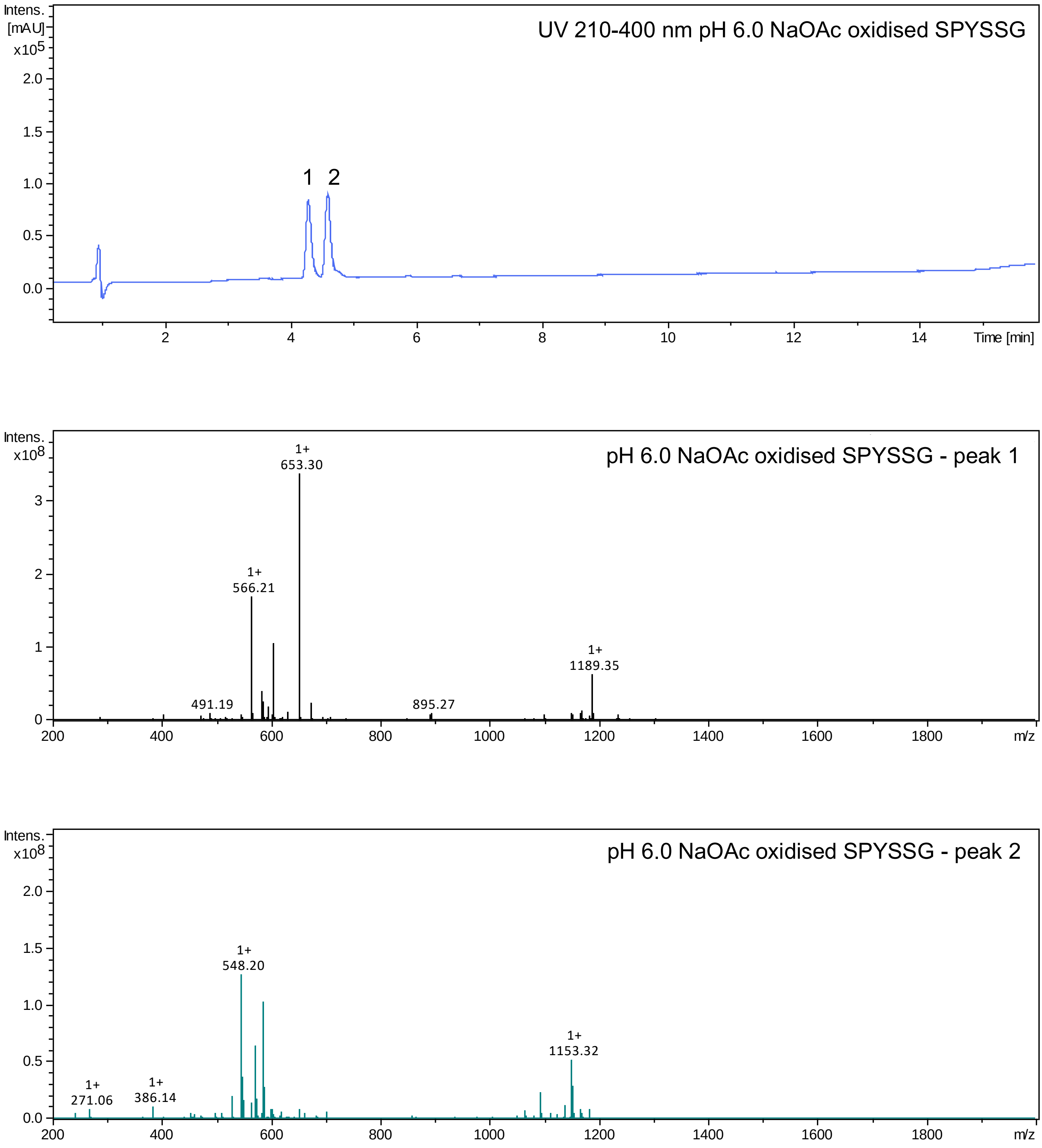

Figure S6. Full LCMS analysis of pH 6.0 NaOAc oxidised SPYSSG

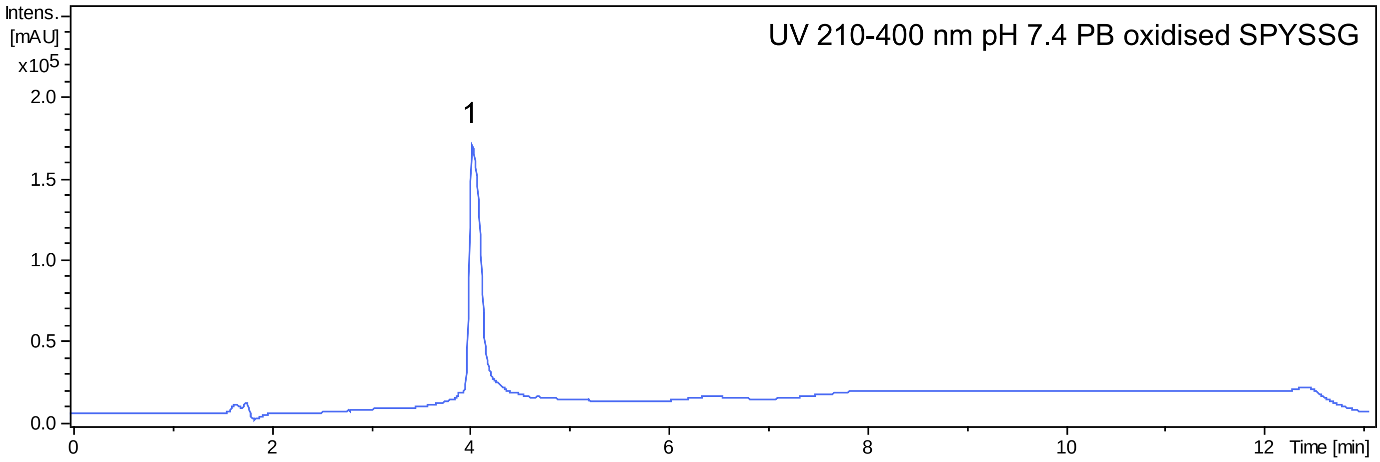

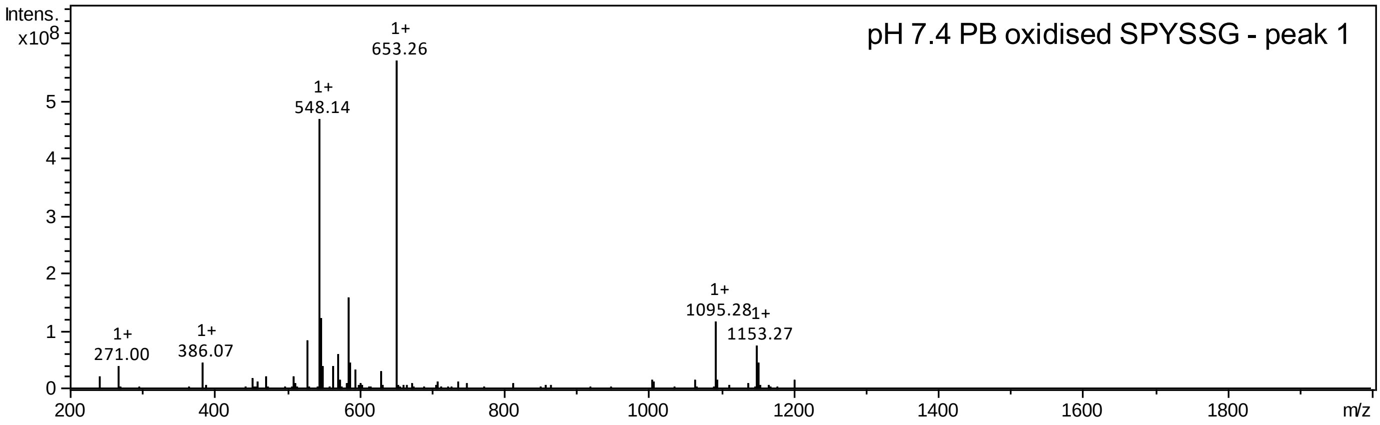

Figure S7. Full LCMS analysis of pH 7.4 PB oxidised SPYSSG

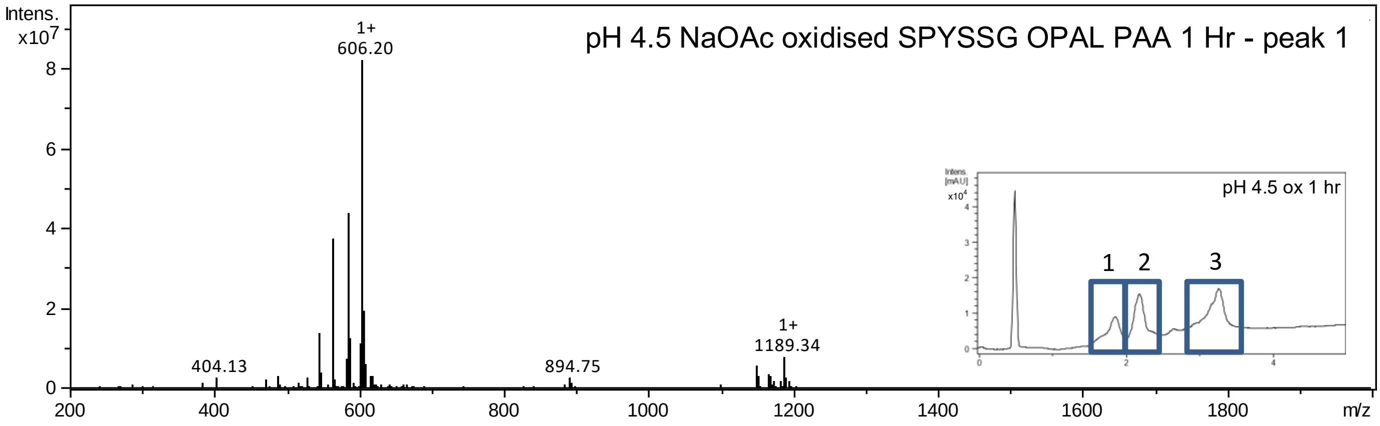

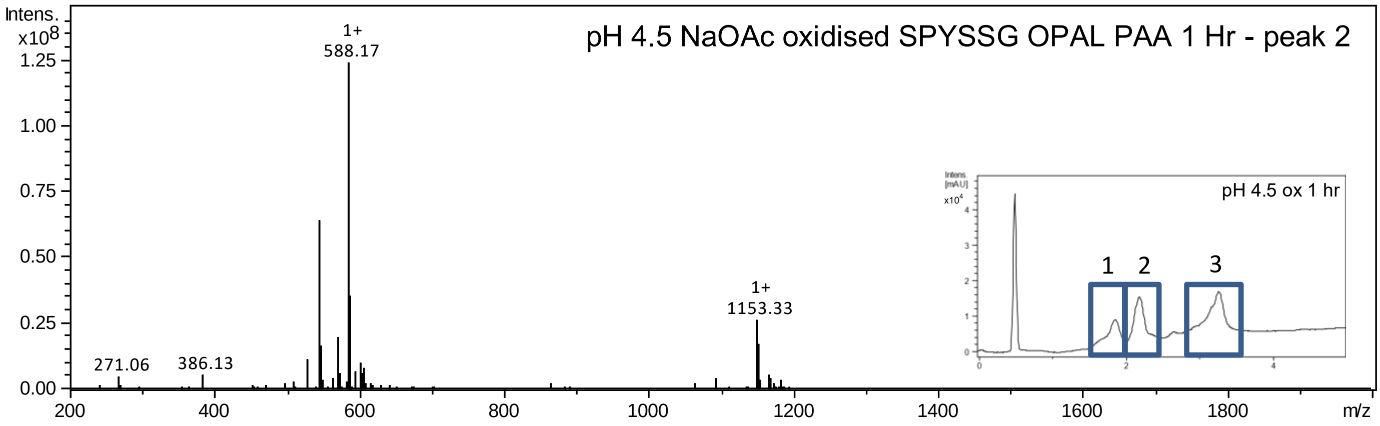

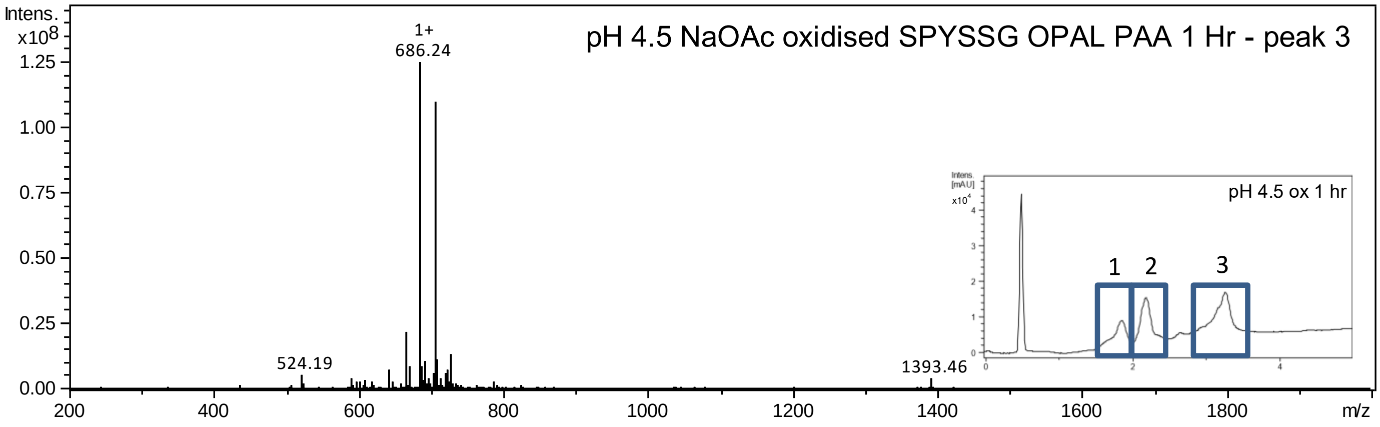

Figure S8. Full LCMS analysis of pH 4.5 NaOAc oxidised SPYSSG OPAL with phenylacetaldehyde (PAA) 1 Hr

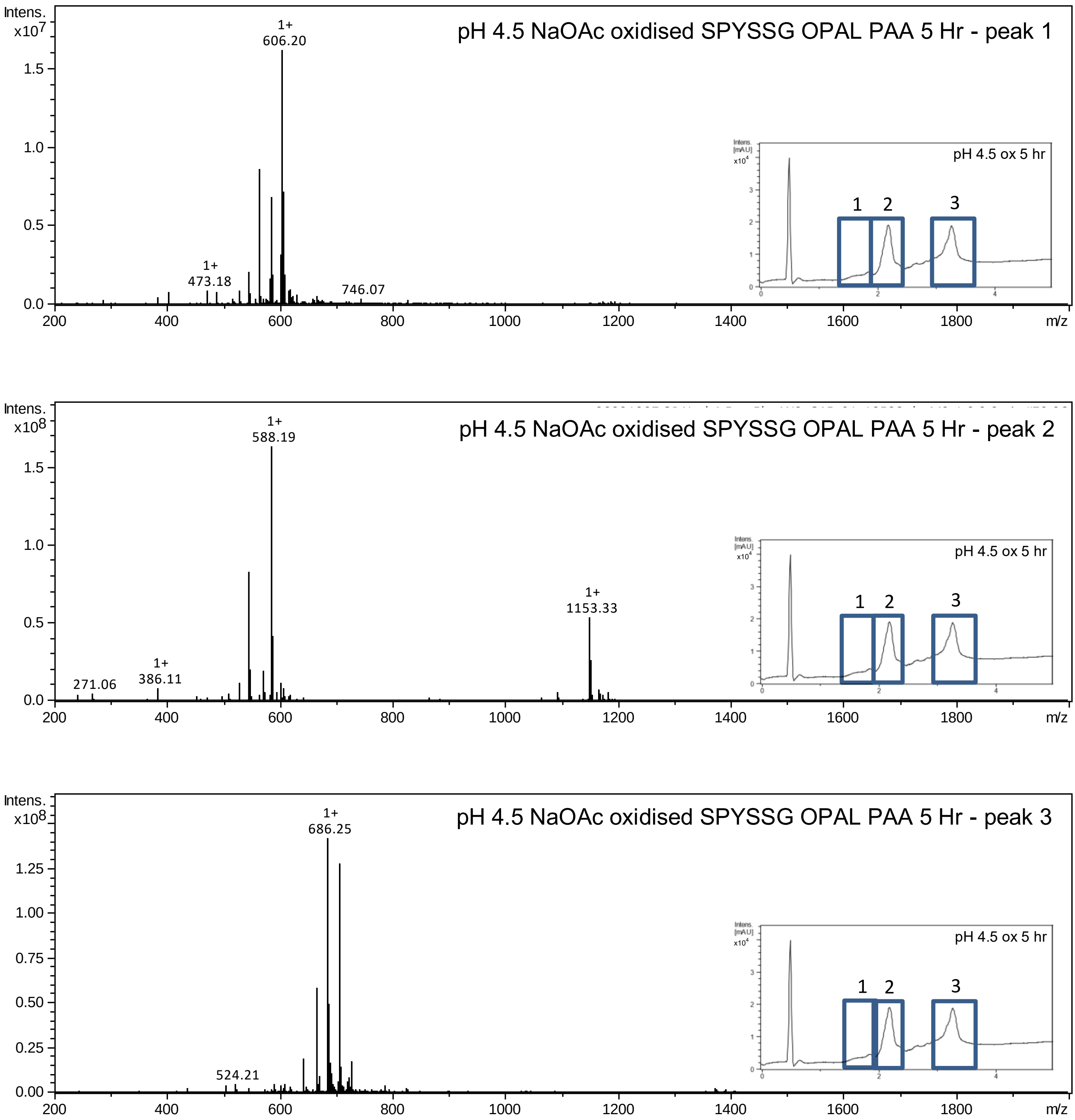

Figure S9. Full LCMS analysis of pH 4.5 NaOAc oxidised SPYSSG OPAL with phenylacetaldehyde (PAA) 5 Hr

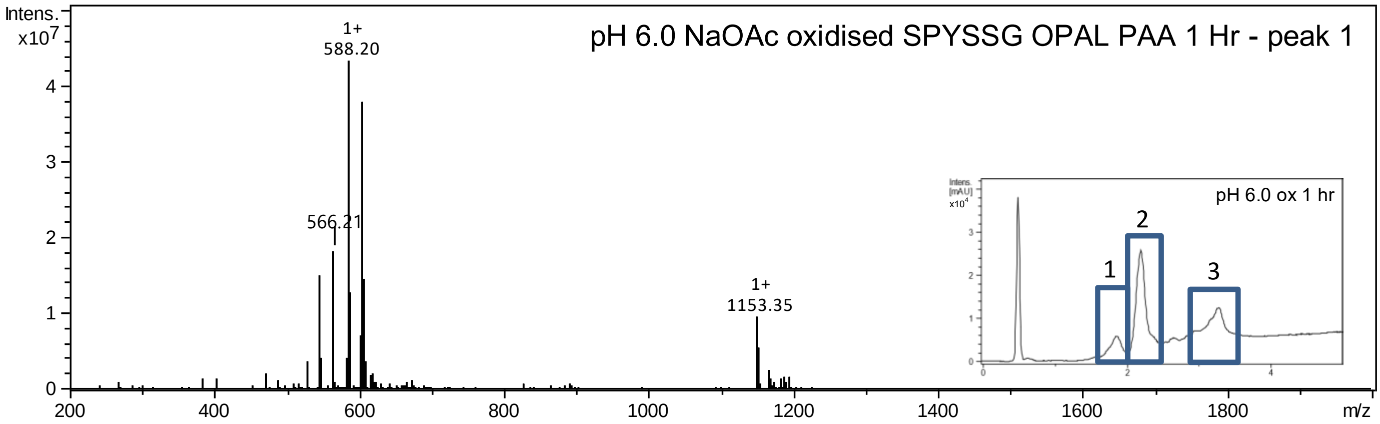

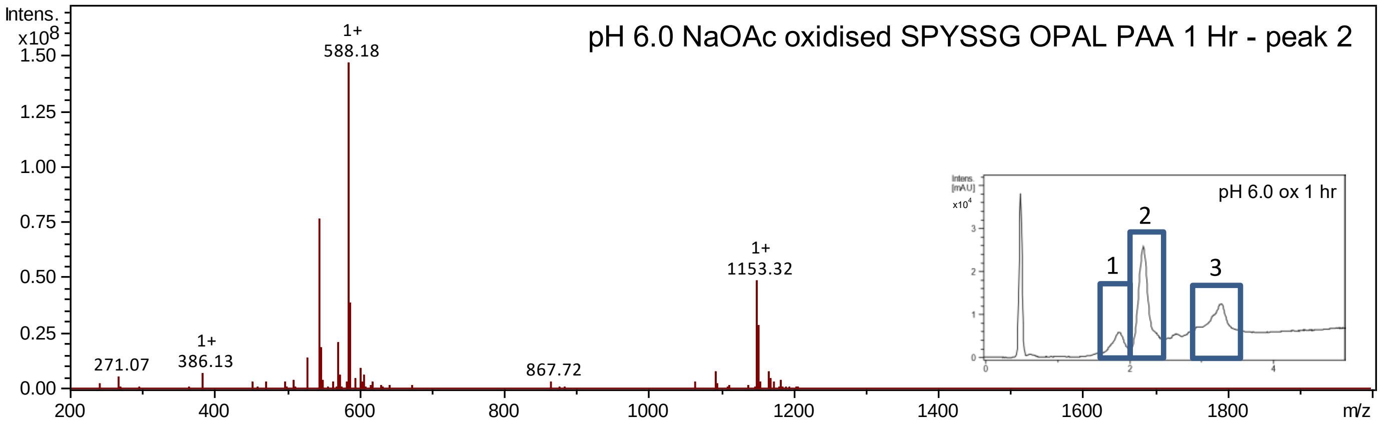

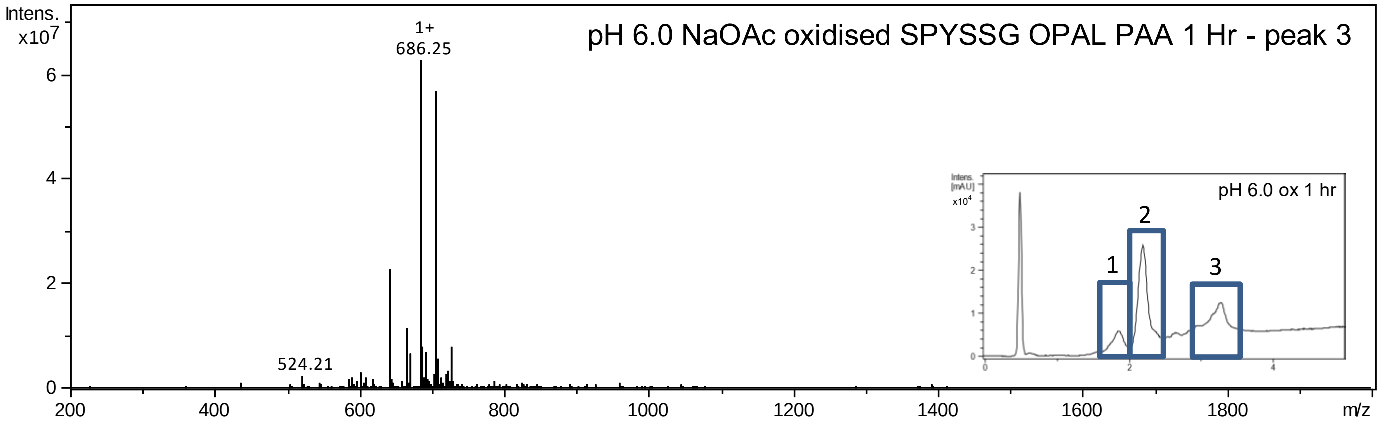

Figure S10. Full LCMS analysis of pH 6.0 NaOAc oxidised SPYSSG OPAL with phenylacetaldehyde (PAA) 1 Hr

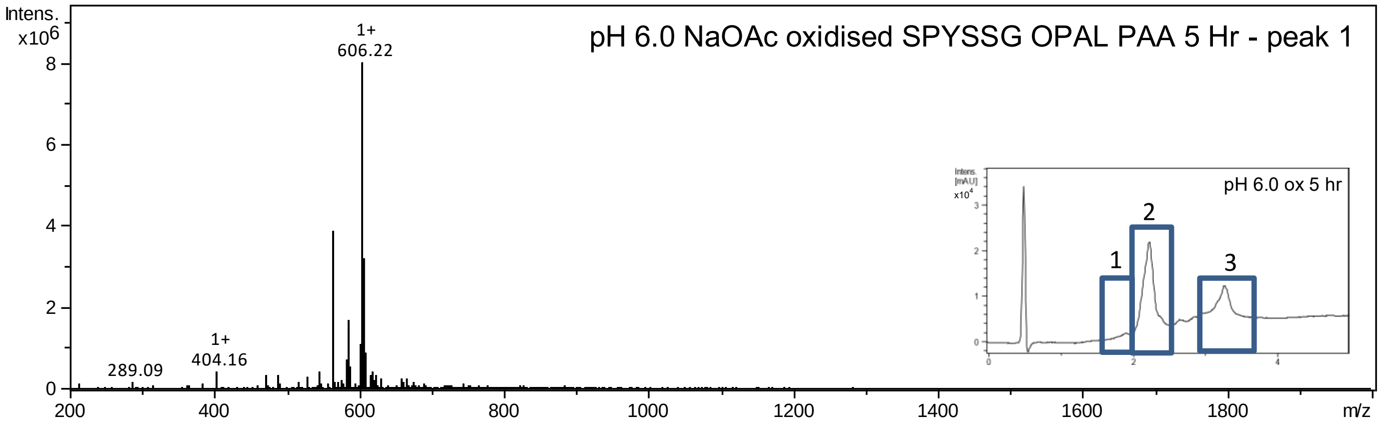

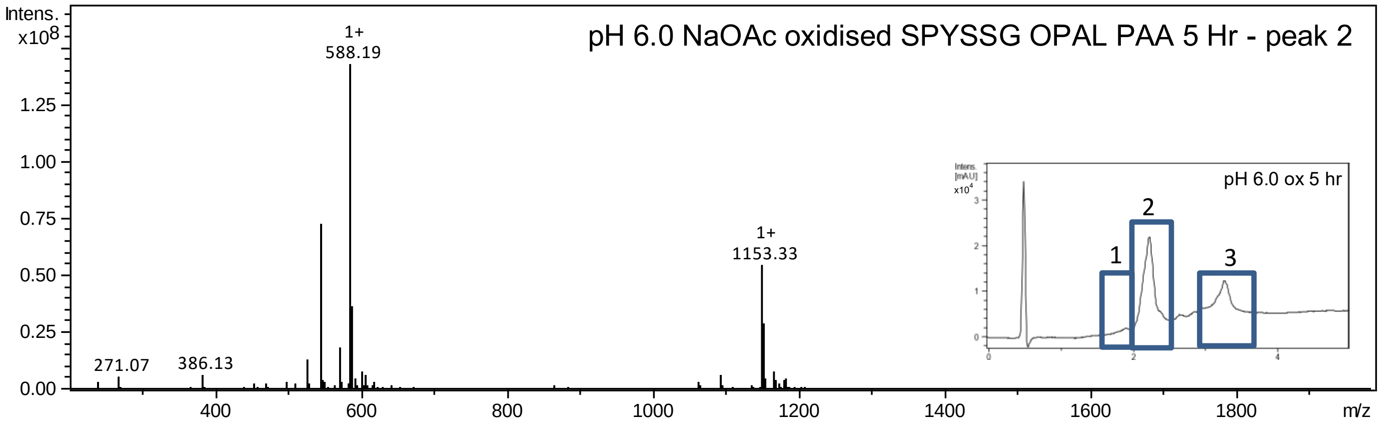

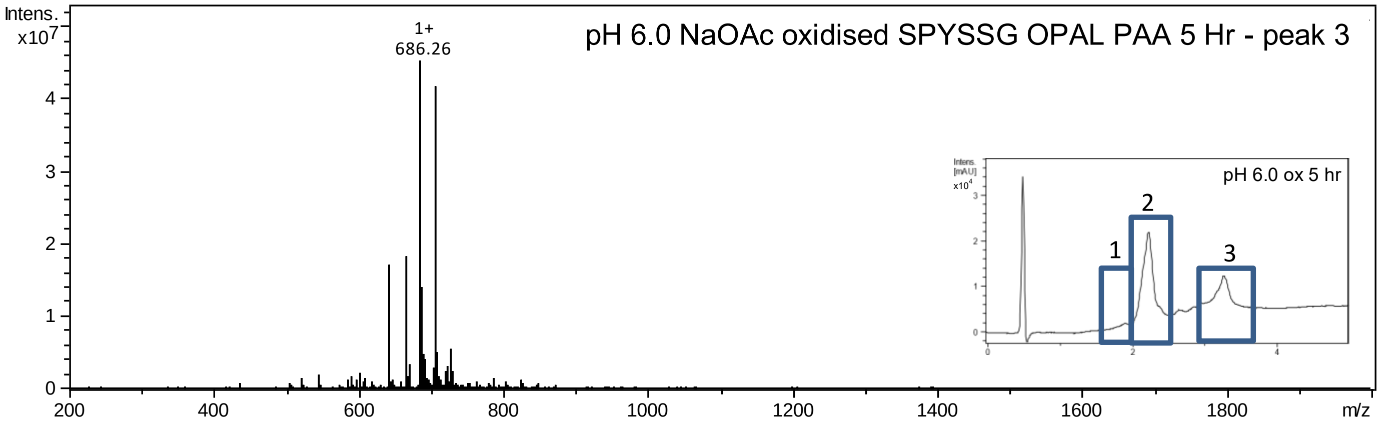

Figure S11. Full LCMS analysis of pH 6.0 NaOAc oxidised SPYSSG OPAL with phenylacetaldehyde (PAA) 5 Hr

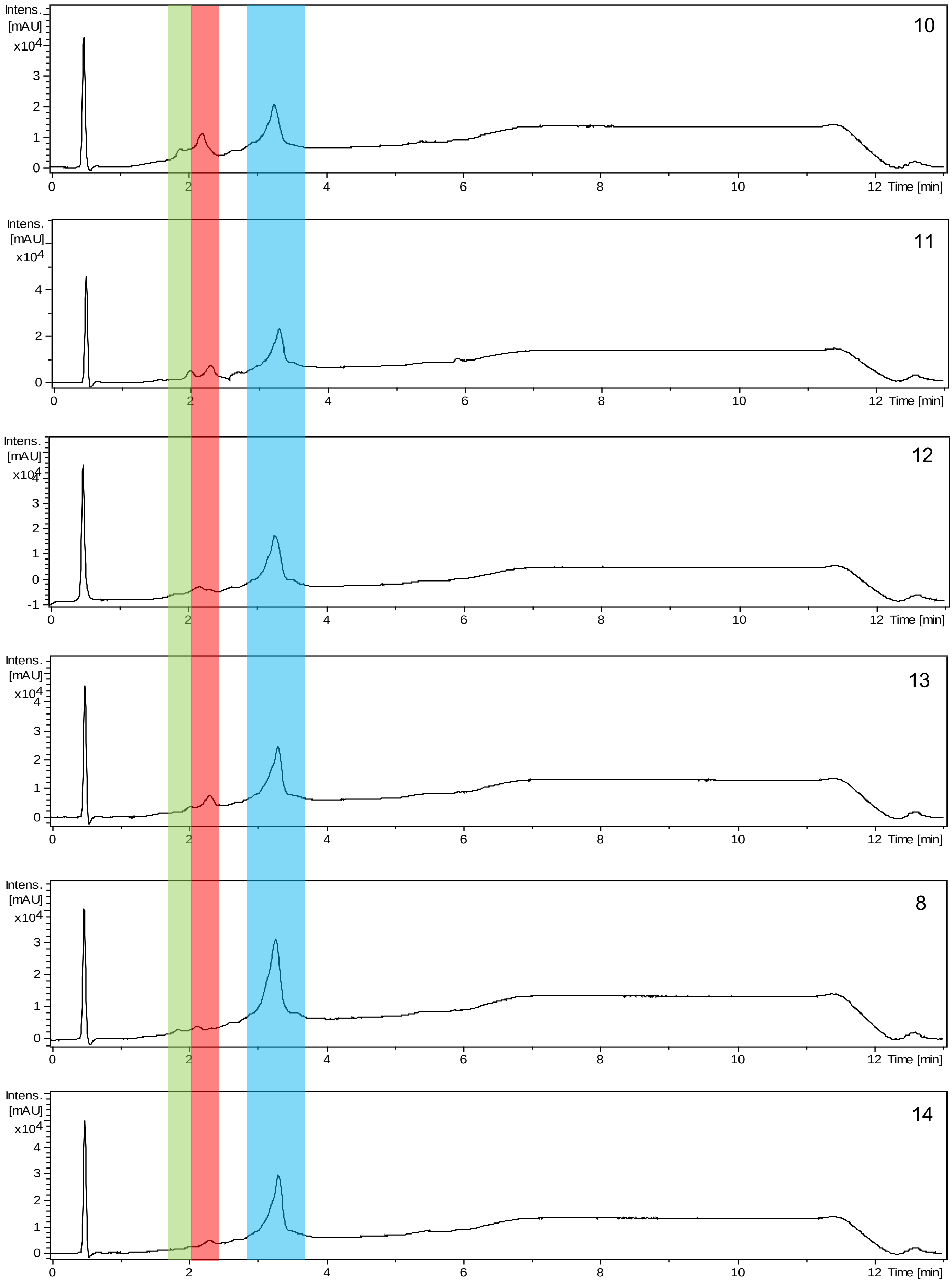

Figure S12. SPYSSG OPAL catalyst (**8, 10-14**) screening UV chromatogram 210-400 nm. Green represents oxidised SPYSSG, red represents cyclised SPYSSG and blue represents OPAL product.

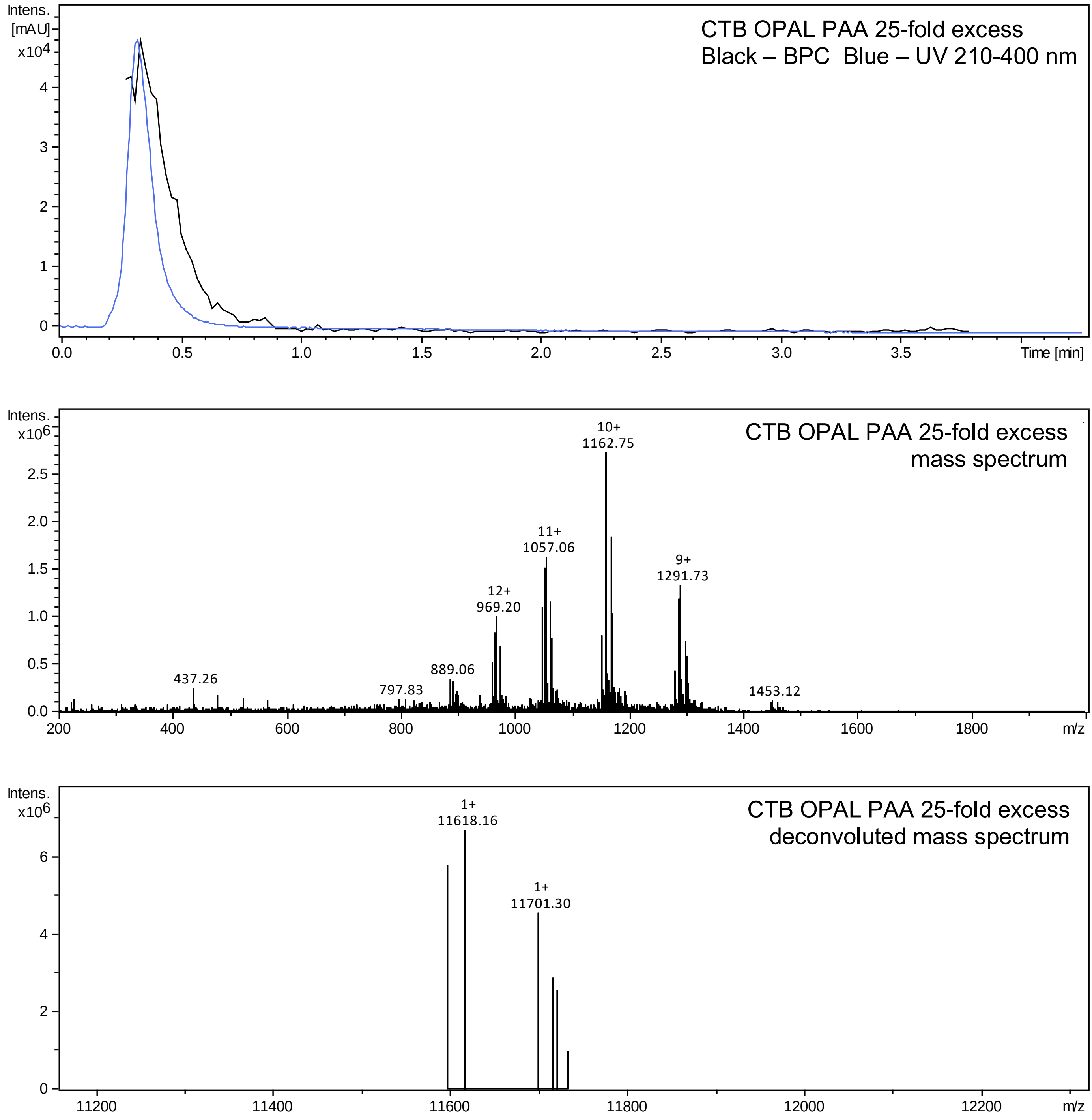

Figure S13. Full protein mass spectra CTB OPAL product **16** using phenylacetaldehyde **7** (PAA) at 25-fold excess

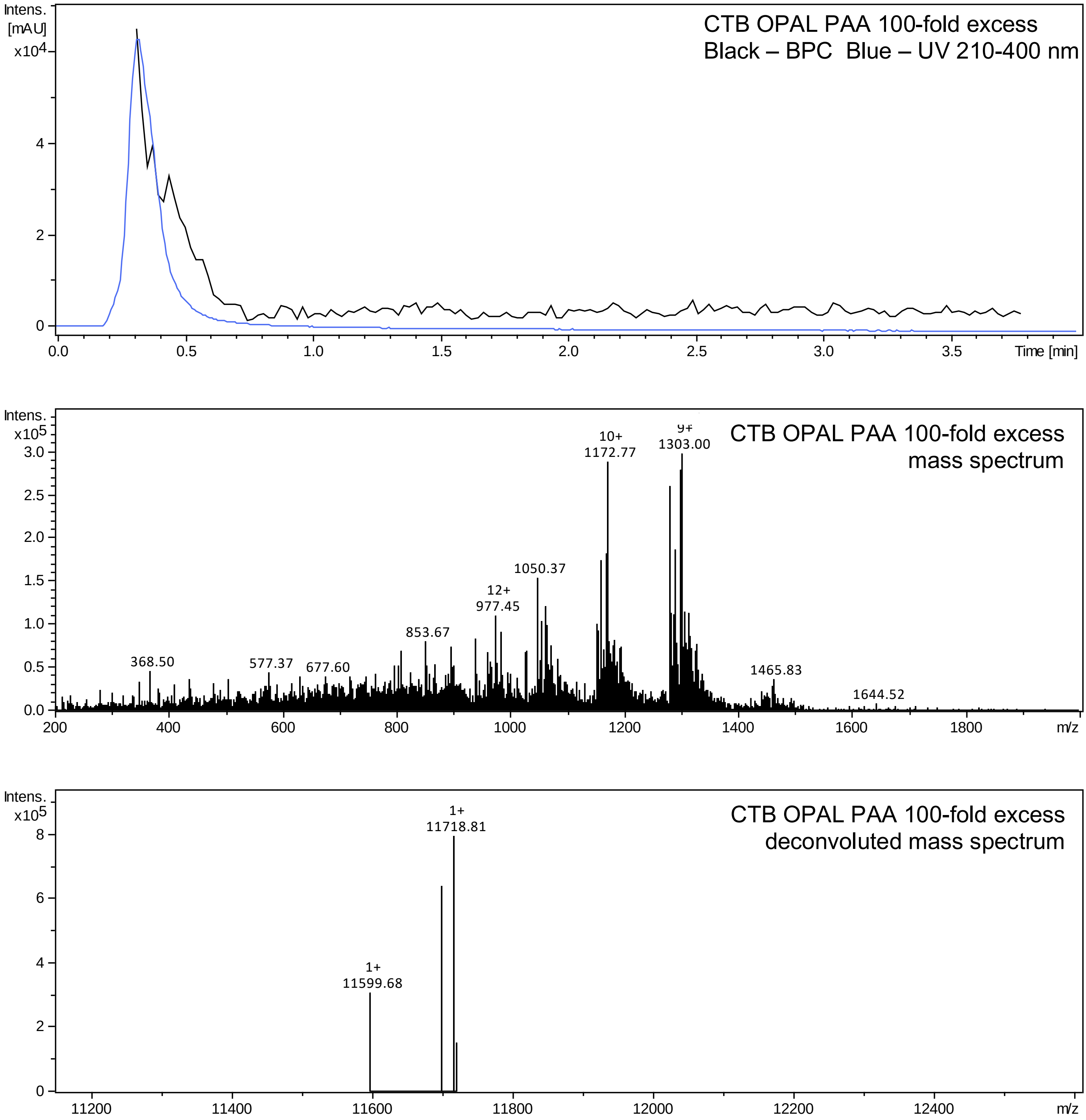

Figure S14. Full protein mass spectra CTB OPAL product **16** using phenylacetaldehyde **7** (PAA) at 100-fold excess

References

1. T. R. Branson, T. E. McCallister, J. Garcia-Hartjes, M. A. Fascione, J. F. Ross, S. L. Warriner, T. Wennekes, H. Zuilhof, W. B. Turnbull, *Angew. Chem. Int. Ed.*, 2014, **53** (32), 8323-8327.
2. N. D. J . Yates, S. Akkad, A. Noble, T. Keenan, N. E. Hatton, N. Signoret and M. A. Fascione, *Green Chem.,* 2022, **24**, 8046-8053.
